# First Bronze Age Human Mitogenomes from Calabria (Grotta Della Monaca, Southern Italy)

**DOI:** 10.3390/genes12050636

**Published:** 2021-04-25

**Authors:** Francesco Fontani, Elisabetta Cilli, Fabiola Arena, Stefania Sarno, Alessandra Modi, Sara De Fanti, Adam Jon Andrews, Adriana Latorre, Paolo Abondio, Felice Larocca, Martina Lari, David Caramelli, Emanuela Gualdi-Russo, Donata Luiselli

**Affiliations:** 1 Department of Cultural Heritage, University of Bologna, via Degli Ariani 1, 48121 Ravenna, Italy; francesco.fontani2@unibo.it (F.F.); elisabetta.cilli@unibo.it (E.C.); a.andrews@unibo.it (A.J.A.); adriana.latorre@studio.unibo.it (A.L.); 2 Department of Neuroscience and Rehabilitation, University of Ferrara, Corso Ercole I D’Este 32, 44121 Ferrara, Italy; fabiola.arena@unife.it (F.A.); emanuela.gualdi@unife.it (E.G.-R.); 3 Centro Regionale di Speleologia “Enzo dei Medici”, via Lucania 3, 87070 Roseto Capo Spulico, Italy; felicelarocca1964@gmail.com; 4 Department of Biological Geological and Environmental Sciences, University of Bologna, via Selmi 3, 40126 Bologna, Italy; sara.defanti2@unibo.it (S.D.F.); paolo.abondio2@unibo.it (P.A.); 5 Department of Biology, Università degli Studi di Firenze, via del Proconsolo 12, 50125 Firenze, Italy; alessandra.modi@unifi.it (A.M.); martina.lari@unifi.it (M.L.); david.caramelli@unifi.it (D.C.); 6 Interdepartmental Centre “Alma Mater Research Institute on Global Challenges and Climate Change (Alma Climate)”, University of Bologna, via Petroni 26, 40126 Bologna, Italy; 7 Gruppo di Ricerca Speleo-Archeologica, Università degli Studi di Bari “Aldo Moro”, Piazza Umberto I 1, 70121 Bari, Italy

**Keywords:** ancient DNA, paleogenomics, human, mitochondrial DNA, archaeology, Italy, Bronze Age

## Abstract

The Italian peninsula was host to a strong history of migration processes that shaped its genomic variability since prehistoric times. During the Metal Age, Sicily and Southern Italy were the protagonists of intense trade networks and settlements along the Mediterranean. Nonetheless, ancient DNA studies in Southern Italy are, at present, still limited to prehistoric and Roman Apulia. Here, we present the first mitogenomes from a Middle Bronze Age cave burial in Calabria to address this knowledge gap. We adopted a hybridization capture approach, which enabled the recovery of one complete and one partial mitochondrial genome. Phylogenetic analysis assigned these two individuals to the H1e and H5 subhaplogroups, respectively. This preliminary phylogenetic analysis supports affinities with coeval Sicilian populations, along with Linearbandkeramik and Bell Beaker cultures maternal lineages from Central Europe and Iberia. Our work represents a starting point which contributes to the comprehension of migrations and population dynamics in Southern Italy, and highlights this knowledge gap yet to be filled by genomic studies.

## 1. Introduction

The genomic variability within the Italian Peninsula is greater today than in any European country, which may suggest that this area played a pivotal role in the peopling of the Mediterranean in the past [1]. Genetic studies based on autosomal and uniparental markers [2,3,4], genome-wide [1,5,6,7,8,9] and whole genome approaches [10] have dissected the clinal variability of the present Italian population, revealing multilayered patterns of prehistorical and historical processes of migration and admixture that occurred throughout the Peninsula. In particular, these studies provided evidence of an early divergence of Italian groups dating back to the Late Glacial period, with further differentiation attributed to Neolithic and Bronze Age migrations [10].

Modern Southern Italian populations have been extensively studied [4,6,7,10,11], highlighting a long series of migration processes and cultural exchanges that influenced this area. High frequencies of maternal lineages from the Caucasus and the Levant were retrieved, which predate the Neolithic and may support the role of this area as a refugium during the Last Glacial Maximum (LGM) [6,12]. A wide genetic Mediterranean “*continuum*” was identified, which links Southern Italy with Crete and the Caucasus following Neolithic and Bronze Age migrations from the Near East. In addition, during the Bronze Age, a non-steppe contribution derived from the Caucasus was detected [7,10].

**Figure 1:**
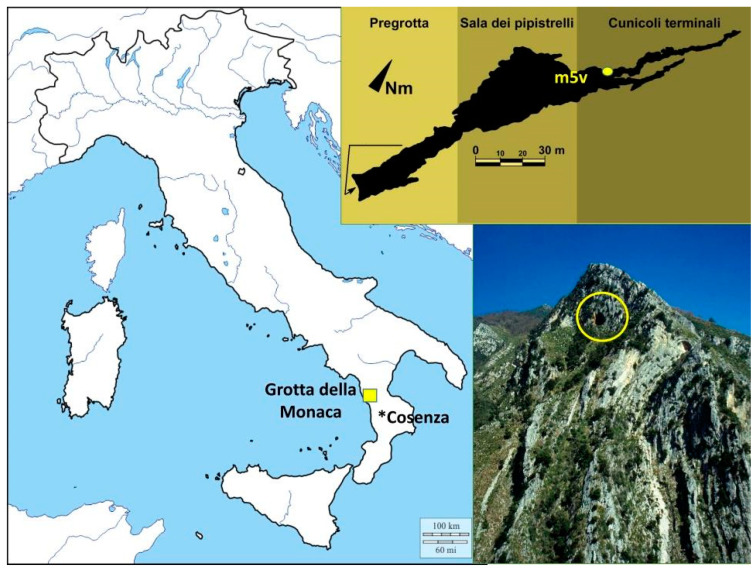
Location of Grotta della Monaca. The entrance of the cave is marked by a yellow circle on the bottom right image. The scheme of the cave with the *m5v* area is represented at the top right.

The Metal Age, in particular, deeply characterized the complex population processes of protohistoric Italy, leaving clear signatures in modern populations [13]. Territorialization spread in both Tyrrhenian and Adriatic populations during the Bronze Age, and dynamism was further promoted following novel social structuring [14]. The emergence of elite groups during the second millennium BC [15] resulted in community organization pivoted around kinship and inherited rank in Southern communities [16]. The Cosenza province (central Calabria) played an important role in the landscape of prehistoric studies of Southern Italy due to this structuring. Evidence of consistent mining activities nearby the Sila Plateau allowed protohistoric Calabria to establish itself as one of the fundamental territories for metallurgy in Italy, and to connect this area in a strong exchange relationship within the lower Tyrrhenian coastal communities [17]. The presence of a Protoapennine culture in many Middle Bronze Age (MBA) Calabrian contexts, such as Broglio di Trebisacce [18], was suggested by Ardesia [19] to be the “Calabrian” Rodì-Tindari-Vallelunga (RTV) horizon, thus underlining the strong interactions and shared cultural patterns between the southern areas and the western islands of the peninsula. Nevertheless, a deep analysis of funerary contexts from protohistoric Southern Italy is essential. The presence of numerous cave sites that have not yet been investigated in the area of Tyrrhenian Calabria prompt the need for a deeper knowledge of the ancient communities of Southern Italy. The northwestern sector of Calabria is rich in cave sites, with over two hundred caves present along the Tyrrhenian coast and inland, such as Grotte di Cirella in Diamante, Grotte di Torre Talao in Scalea, Grotta della Madonna in Praia a Mare, Grotta del Romito in Papasidero, Grotte di Sant’Angelo in Cassano allo Ionio and Grotta di Donna Marsilia [20]. Therefore, paleogenetic studies from this area, in particular, are warranted to investigate early population dynamics. To complement genetic studies on modern populations, ancient DNA (aDNA) studies have the potential to provide precise insights into early heritage and migrations, coupled with sociocultural aspects, of past societies. However, aDNA studies in Southern Italy are limited to prehistoric [21,22] and Roman [23] Apulia. Hence, we aimed here to produce mitogenome data on Bronze Age communities from Calabria, in Southern Italy, to provide new information on population dynamics from this understudied area and to contextualize them with archaeological and anthropological evidence. To achieve this, we explored the remains of a MBA cave burial in Tyrrhenian Calabria, Grotta della Monaca (Figure 1).

## 2. Materials and Methods

### 2.1. Archaeological Context and Human Remains

Grotta della Monaca is a karstic cave located in Sant’Agata di Esaro at 600 m above sea level, close to the Esaro river (Cosenza, northwestern Calabria, Southern Italy) (Figure 1). The cave has a long human history [20,24,25] due to mining activities that began during the Upper Paleolithic period and intensified during the Late Neolithic/Early Chalcolithic period [26]. The cave partially changed its role during the MBA, when the deepest areas were used as burial grounds [27,28]. Archaeological investigations conducted between 2003 and 2010 recovered a mass grave with numerous human skeletal remains in a niche of the area of Cunicoli terminali (hereafter *m5v*). Collective burials in a natural cave were a common and uninterrupted ritual in protohistoric Southern Italy, particularly in the coastal sites facing the Tyrrhenian Sea [29]. From the Early Bronze Age (EBA), this funerary practice was reserved for high-status individuals [30]. Fragmented pottery and funerary artifacts evidence a link between the burials in Grotta della Monaca and the so-called Apennine culture [31], which was an archaeological complex that was widespread in the Italian Peninsula and Sicily during the second half of the second millennium BC. The remains found in the *m5v* context represent at least 24 different commingled individuals (File S1 and Table S1), and were in a poor state of preservation due to several taphonomic factors [32]. The osteological remains buried in *m5v* of Grotta della Monaca were examined by traditional anthropological methodologies at the Laboratory of Archaeo-Anthropology and Forensic Anthropology of the University of Ferrara (See File S1 for details about the methods used and the anthropological data acquired).

Radiocarbon analysis of the human bone samples was conducted at CEDAD (Center of Applied Physics, Dating and Diagnostics, University of Salento, Lecce, Italy). Six out of seven samples were successfully dated and were assigned to the MBA period. Calibrated and uncalibrated dates are summarized in Table S2. 

### 2.2. Experimental Procedure

Ancient DNA analysis was performed on seven individuals (Table S3 and Figure S1) from the *m5v* mass grave. Due to the poor preservation of the remains, the only petrous bone available (preferred because it is DNA-rich [33,34]) was associated with individual 24. Thus, four shafts of long bones (GdM5, GdM6, GdM7, GdM12) and two cranial fragments (GdM11, GdM14) were sampled along with the petrous bone (GdM24). The samples were selected based on the preservation state, and took into account the bone elements used to calculate the minimum number of individuals (MNI), to make sure we sampled different individuals. Analysis was conducted in the ancient DNA Laboratory (aDNA Lab) of the Department of Cultural Heritage, University of Bologna (Ravenna, Italy). Post-PCR procedures were performed in a separate laboratory, and negative control was carried out alongside each experiment to confirm the absence of intra-laboratory contamination. All materials and instruments used were DNA-free and decontaminated before use with bleach and/or DNA ExitusPlus™ (AppliChem GmbH, Darmstadt, Germany) and researchers followed high-sterility protocols according to ancient DNA authenticity criteria [35,36,37,38]. 

Specimens were first cleaned with sterile scalpels to remove sediments and calcareous concretions, and 1–2 mm of the outer layer of bones was abraded with circular drilling discs. Bones were decontaminated for 1 h under UV irradiation, and 140 to 340 mg fragments of the bone specimens were pulverized, while 255 mg of bone powder was collected from petrous bone by directly drilling the densest region of the cochlea.

### 2.3. Ancient DNA Extraction

DNA from the samples was extracted following a two-day silica spin-column protocol [39,40] with slight modifications, as in Cilli et al. [41]. Samples were digested in a rotator at 37 °C overnight in 3000 µL solution composed of 2700 µL EDTA, 37.5 µL Proteinase K and H_2_O. Supernatant was centrifuged and transferred with PB binding buffer (Qiagen, Hilden, Germany) into silica columns (Roche-High Pure Viral Nucleic Acid Large Volume Kit, Roche, Basel, Switzerland). After two washing steps with PE buffer (Qiagen), DNA was eluted in 50 µL of EB buffer (Qiagen).

### 2.4. Library Preparation and Enrichment

Double-stranded library preparation was performed according to Carøe et al. [42]. Libraries were indexed with Illumina sequencing adapters and no enzymatic damage repair was carried out in order to preserve patterns of ancient DNA fragments. A qPCR quantification was performed prior to indexing, in order to assign correct amplification cycles to each library and to reduce the formation of heteroduplex structures. A total volume of 30 µL from each library was split in 2/3 aliquots and used for indexing PCR. PCR indexing products were purified with MinElute PCR Purification kit (Qiagen), eluted in EBT buffer (10 mM Tris-Cl, pH 8.5 and 0.05% Tween-20) and analyzed on Agilent 2100 Bioanalyzer (Agilent, Santa Clara, CA, USA) to produce qualitative and quantitative estimations of the libraries. 

Indexed libraries were equimolarly pooled to a total of maximum 2 µg and enriched for human mitochondrial DNA (mtDNA) for the bead-capture method using long-range PCR products, according to Maricic et al. [43]. The captured libraries were quantified using Agilent 2100 Bioanalyzer and sequenced on Illumina MiSeq platform at the Advanced Genomic Centre of the University of Florence. The sequencing was run as paired-end with 75 × 2 + 8 + 8 cycles.

### 2.5. Bioinformatic Analysis

Raw reads were first visualized with FastQC [44], and adapter sequences were filtered using AdapterRemoval v2.3.0 [45]. Fragments shorter than 30 bp and with a ‘*minquality < 25*’ (PHRED values) were discarded, and paired-end reads with overlaps of at least 10 bp were collapsed into single sequences. Filtered reads were mapped against the revised Cambridge Reference Sequence (rCRS, NC_012920.1) [46] with BWA v.0.7.17-r188 [47]. Command ‘*aln*’ was set with -n number to 0.01, seed-length option deactivated and -o value at 3 for tolerating a higher number of gaps in the alignment. The generated .sai file was aligned against the reference fasta file with ‘*samse*’ command, and then converted to .bam files and sorted by leftmost coordinate using SAMtools v1.10 [48] ‘*view*’ and ‘*sort*’ commands. The Picard tool MarkDuplicates [49] was used on the .bam files to mark and remove PCR duplicates, and reads were locally realigned through Genome Analysis Toolkit tools’ [50] ‘*RealignerTargetCreator*’ and ‘*IndelRealigner*’ commands, to minimize the number of mismatches around the indels that may be easily mistaken as SNPs.

Data authenticity and post-mortem damage were investigated through MapDamage v2.0.8 [51] and a new rescaled .bam file was generated with the ‘*rescale*’ option, that downscales the quality scores at positions likely affected by damage patterns. Damage patterns at the 5′ and 3′ ends of the reads were also computed with contDeam, a tool provided within the Schmutzi package [52], and present-day human contamination estimates were performed with Schmutzi, using a non-redundant database of 256 mitogenomes available in the software package. Results are summarized in Table S3. Mitochondrial genomes were called using Schmutzi, and variants were then investigated using GATK *‘HaplotypeCaller’* and filtered with *‘VariantFiltration’* command, storing only detected variants with quality ‘*QUAL20*’ (≥20) and depth of coverage ‘*COV3*’ (≥3). Diagnostic variants were checked and verified with snpToolkit v2.2.3 [53], and only variants with ratio −r 0.9 were used to determine the haplogroup. 

### 2.6. Phylogenetic Analyses

Phylogenetic analyses were performed on the complete mitogenome of individual GdM24, while GdM7 was only analyzed for haplogroup assignment. The other samples were not included due to inconsistencies in the data (Table S3). The mtDNA haplogroup assignment was predicted using both Haplofind [54] and HaploGrep [55] software, and checked manually according to PhyloTree build 17 [56]. The consensus sequence obtained for GdM24 was then compared to a reference dataset of 97 ancient individuals extracted from the literature (Table S4). In addition, 13 published mitogenomes from ancient African and European samples that supply basal lineage information for phylogenetic reconstruction were included in the analyses (Table S5). The RSRS and rCRS reference sequences were also added for comparison purposes in the Network analysis. 

Sequence alignment was performed with the DNA Alignment software [57] and was visually confirmed. The poly-C and AC-indels at 303–315, 515–524, 573–576 and 16,180–16,193 positions, as well as the nucleotide position 16,519, were excluded from phylogenetic analyses [58]. A Median Joining Network was calculated by using Network v.10.2 [59] with no pre- or post-processing steps. Furthermore, a phylogenetic tree, based on the same multi-alignment dataset after retaining unique sequences, was reconstructed with BEAST v1.8.0 [60] by using reference ancient dated samples as calibration points (Table S5). The best substitution model for the dataset was tested with Mega 5.2 [61], resulting in the Hasegawa–Kishino–Yano model with fixed fraction of invariable sites and gamma distributed rates (HKY + I + G). We ran BEAST analysis with 200,000,000 MCMC generations, and sampled every 2000 iterations by testing different combinations of clock models (i.e., Strict clock vs. Uncorrelated Relaxed Lognormal-ULN clock) and tree models (i.e., Constant Population Size vs. Bayesian Skyline). Chain convergence was assessed with Tracer v 1.6, resulting in Effective Sampling Size (ESS) values higher than 200 for all the parameters and in all the tested model combinations. Evaluating the maximum likelihood estimates (MLE) for the four combinations of clock and tree models further revealed the best support for the Relaxed ULN clock model and Bayesian Skyline tree model; therefore, this combination was chosen for the final step of analysis. The Maximum Clade Credibility tree was calculated using TreeAnnotator v1.8.0 by discarding the first 20% of iterations as burn-in. The resulting phylogenetic tree was graphically represented with FigTree [62].

## 3. Results

Six human bone fragments and one petrous bone from the *m5v* burial area of Grotta della Monaca were analyzed for ancient mtDNA on the Illumina MiSeq platform. Five out of seven samples contained low concentrations of DNA (Table S3) and were not further analyzed. For the sample GdM24, we obtained 10,471 mapping reads with quality ≥30, which resulted in a mean coverage of 34X, and 100% of positions covered at least 3-fold. GdM7 contained 550 reads mapped with quality ≥30, resulting in a mean coverage of 2X, and 91% of positions covered at least 1-fold. Analysis of the read datasets with MapDamage v2.0.8 revealed miscoding lesion distribution patterns typical of ancient DNA in both samples (Figure S2). The presence of positions’ specific substitutions increases at the ends of the reads, with an average frequency of 28.67% (GdM7) and 45.46% (GdM24) of C-to-T substitution at the first base at 5′, and similar average frequency of 33.80% (GdM7) and 42.85% (GdM24) G-to-A substitution at 3′ ends. The average fragment length is around 56.49 for GdM24 and 77.33 for GdM7. These data are consistent with the antiquity of the samples and the environmental conditions of the site.

The GdM24 mitochondrial genome was assigned with 85.63% accuracy (Haplogrep) and 1.0 score (Haplofind) to the H1e mitochondrial subclade, based on the relevance of the eleven diagnostic variants detected (263G, 1438G, 3010A, 4769G, 5460A, 6779G, 8860G, 15326G, 16172C, 16224C, 16519C). Regarding GdM7, haplogroup assignment was inferred on the partial sequence, evaluating the presence of diagnostic variants (263G, 1438G, 2626C, 4769G, 8860G, 16304C, 16310A). This appears to belong to the mitochondrial subclade H5, with 79.95% accuracy on Haplogrep and 1.0 quality score on Haplofind, respectively. However, due to the low coverage and incompleteness of the mitogenome, GdM7 was not included in the subsequent phylogenetic analysis.

**Figure 2:**
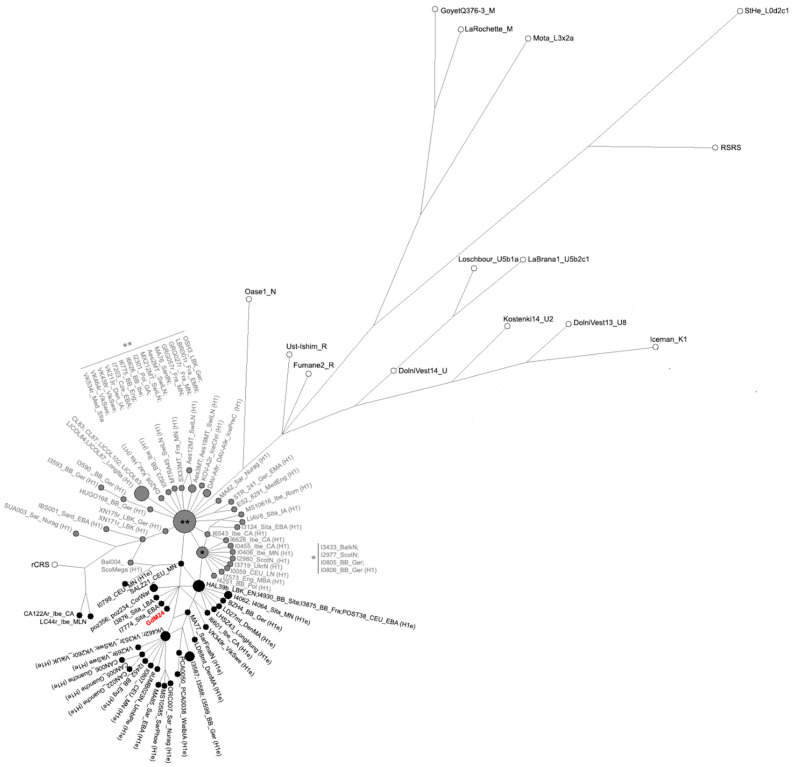
Median Joining Network based on the complete GdM24 mitogenome (shown in red) compared to a dataset of ancient reference (in white), H1 (grey) and H1e (black) mitogenomes (see Table S4 for dataset).

**Figure 3:**
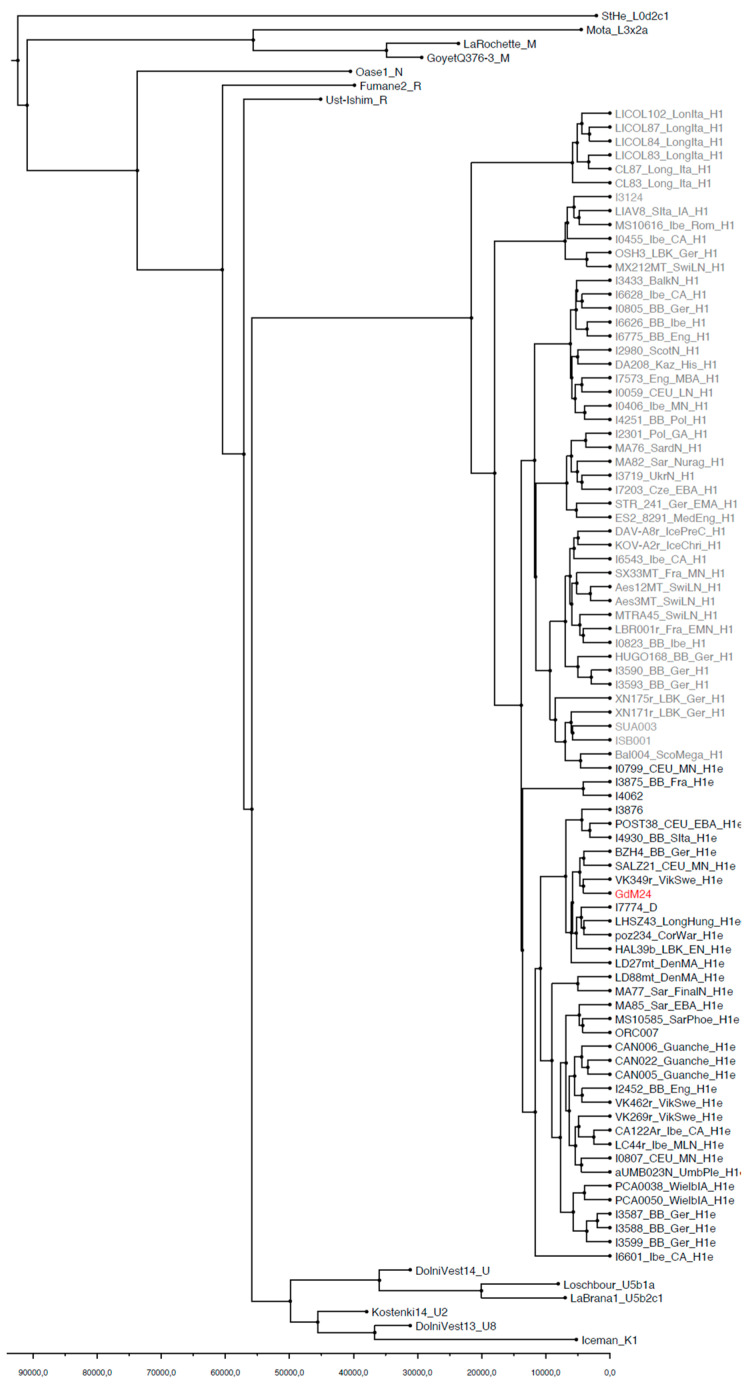
Phylogenetic tree reconstructed with BEAST using dated ancient reference samples as calibration points (Tables S4 and S5).

Phylogenetic reconstruction performed with both Median Joining Network (MJN) and Bayesian Evolutionary Analysis of Sampling Trees (BEAST) confirmed the attribution of GdM24 to the mtDNA haplogroup H1e. Accordingly, the sample indeed clusters with the other H1e samples extracted from the literature, within a clade that splits up after H1 and the other basal-considered lineages (Figure 2 and Figure 3). A star-like pattern characterized both H1 and H1e haplotypes in the Network analysis (Figure 2). In particular, the GdM24 sample branches, along with two Sicilian Bronze Age samples (I3876 and I7774 [63]) from a median vector, originated from the H1e basal node which contains samples from Middle/Late Neolithic and Bronze Age cultures from Central Europe, and a Bell Beaker sample from Southern Italy (I4930 [63]). The same clustering pattern was consistently confirmed in the BEAST reconstructed phylogenetic tree (Figure 3), with the branch leading to the GdM24 sequence, particularly, dating at 4125 years BP (95% HPD: 2301–6332).

## 4. Discussion

Excluding Sicily and Sardinia that experienced different processes of migration and admixture and were covered by several paleogenomic studies [63,64,65,66,67,68,69], Bronze Age Southern Italy has not been studied with an aDNA approach. In this study we attempted analysis of the mitochondrial DNA variation of seven MBA samples from Grotta della Monaca cave, located in Calabria, Southern Italy, with the aim of filling the gap in the contribution of ancient genetic data to the population dynamics of this peculiar area. This is of particular interest and importance considering the key role that the Italian Peninsula played in past demographic processes, as evidenced by the wide variability in genomic diversity in present-day Italian populations [1].

Despite the poor preservation of the remains, the hybridization capture method allowed us to retrieve and reconstruct the complete mitochondrial genome of individual GdM24, and partially reconstruct the mitochondrial genome of individual GdM7. These are the first attested ancient molecular data from the Calabria region, as highlighted by a recent review of the state-of-the-art European paleogenomic data [70]. Unsurprisingly, GdM24 was the only sample for which the petrous bone was available from this burial area. Previous studies suggest that this skeletal element preserves the endogenous DNA better than other bones or tissues [33,71]. Thus, this type of sample is confirmed as fundamental in the recovery of ancient endogenous DNA from particularly difficult contexts, such as those at low latitudes. 

The two samples for which we obtained complete mitogenomes are both assigned to mitochondrial macrohaplogroup H. Nowadays, this is the most widespread in Europe, encompassing over 40% of the total mtDNA variation, and could be considered as the quintessential Eurasiatic marker. Previous studies have shown how haplogroup H arrived in Europe from the Near East before LGM (∼22,000 BP), survived in south western glacial refugia and then colonized Central and Northern Europe [72,73]. In particular, GdM24 was assigned to mitochondrial sublineage H1e, and GdM7 to H5. 

Among the subclades, H1 is the most frequent in modern-day Europeans, followed by H3 and H5 [74]. When compared to other present-day European and Middle Eastern populations, haplogroup H1 shows frequency peaks in the Franco-Cantabrian region, declining from the west towards Eastern and Southern Europe [75]. Taking into account both the distribution and the coalescence age of this subhaplogroup (9.3–12.8 kya; [76]), it has been hypothesized that it could be correlated with a Late Glacial re-expansion of populations from the Franco-Iberian refugial areas at the end of the Ice Age, from ∼15 kya, or at the end of the Younger Dryas, ∼11.5 kya [77,78,79,80,81]. Along with H3, H1 subclade was also proposed as a marker for a Bell Beaker complex expansion originating in Iberia [72]; however, a recent genome-wide study on ancient genomes does not support migration as an important mechanism of the spread of this culture between Iberia and Central Europe [66]. Ancient DNA evidence also highlighted the earliest presence of the H1e subhaplogroup in Neolithic samples from Hungary (∼7000 BP) [82] and Germany (∼6100 BP) [72]. In Italy, the H1e subhaplogroup was retrieved in Sicily from two Middle Neolithic samples (∼6800 BP), an Eneolithic (∼4700 BP) and an EBA sample (∼4000 BP) [63]. In the present study, phylogenetic analyses of GdM24 highlighted the proximity of this sample, among others, with two Sicilian Bronze Age samples (I3876 and I7774 [63]) recovered in Marcita and Contrada Paolina necropolises, respectively. 

Regarding H5, this subhaplogroup shows a coalescence time of ∼10.7–17.1 kya [76]. There is no agreement about the place of origin of H5; hypotheses have been made about eastern Mediterranean [74,80] or south-west European origin, where it has expanded after the Ice Age, with several possible dispersal routes [76]. However, the dynamics that led to the diffusion of this haplogroup are not yet clearly understood. The oldest evidence of H5 lineage was retrieved in Anatolia (∼8200 BP) [83] and Bulgaria (∼7600 BP) [84]. A migration with a center of origin in the eastern Mediterranean, that would have carried this lineage into Italy and, to a lesser extent, the western Mediterranean, until northern and western Iberia, has been hypothesized [80]. 

The mitochondrial haplogroups found in present-day Calabria ([7], personal data) include all of the haplogroups that have been found in the ancient individuals analyzed here (H1e and H5). None of these are unique to the ancient Southern Italian populations and, therefore, cannot be taken as indisputable proof of local population continuity. Nonetheless, they deserve to be more deeply analyzed at the nuclear level to obtain more information about the ancestry and migration patterns of this area.

In the neighboring Sicily, past population dynamics are better understood due to sampling conducted in two projects [63,66] that detected ancestry typical of early European farmers as a mixture of Anatolia Neolithic and Western Hunter-Gatherer, and no evidence of steppe ancestry in Middle Neolithic samples. They found evidence of steppe ancestry in the EBA, by around 2200 BC, with the forming of a clade with EBA Mallorca, suggesting that the population may harbor ancestry most plausibly from Iberia, with a scenario of west-to-east gene flow. Fernandes et al. [63] retrieved Iranian-related ancestry in Sicily, from the MBA (1800–1500 BC) and Late Bronze Age (LBA), which was widespread among the Aegean by the MBA, along with the Mycenaean cultural expansion or earlier.

The active role of Tyrrhenian Calabria in cultural connections between eastern Sicily, the Aeolian Islands and the Adriatic environments in protohistoric times have been highlighted [85,86,87,88,89]. These connections are demonstrated by the chrono-typological study of pottery, and reflect strong relations between central-southern Calabria, the Aeolian Islands and the northern part of Sicily [90]. However, the so-called Palma Campania *facies* that identifies the MBA contexts of southwestern Italy still generates uncertainties, especially in Tyrrhenian Calabria, due to the lack of in-depth knowledge of the archaeological contexts, and the absence of ancient genomic data. In light of this evidence, our data represents a baseline for a deeper comprehension of the population pattern of prehistoric and protohistoric Southern Italy.

In the area of Tyrrhenian Calabria, the presence of over two hundred cave sites with attested past human frequentation, not yet investigated, prompts the need for a deeper knowledge of the ancient communities of Southern Italy. Future studies, focused on genome-wide data from this peculiar area, could clarify migratory and demographic processes that took place in the prehistory and protohistory of the Southern European continent, provide information about the ancestry of individuals through time and allow the study of intrinsic differences in migratory behavior related to sex-biased processes, adaptation and admixture. 

## Data Availability

Analyzed data for GdM24 sample are available on NCBI GenBank (Accession number: MW853672). Raw data for all samples have been uploaded on ENA database (Study accession number: PRJEB44125).

## Supplementary Materials

The following are available online at https://www.mdpi.com/article/10.3390/genes12050636/s1, File S1: Archaeological context and anthropological analyses, Figure S1: specimens used for molecular analyses, Figure S2: MapDamage plot, Table S1: Summary of biological profile and pathologies in individuals from Grotta della Monaca involved in genetic analyses, Table S2: Radiocarbon dating and calibration executed on human samples from Grotta della Monaca, Table S3: Summary of the genomic analyses, Table S4: Ancient samples belonging to H1 and H1e haplogroups used in phylogenetic analyses; Table S5: Dataset of reference ancient sequences used as tip calibration points in the BEAST analyses.
