# Supplementary material for: First Bronze Age Human Mitogenomes from Calabria (Grotta Della Monaca, Southern Italy)
Source: Genes (Basel). 2021 Apr 25;12(5):636. doi: 10.3390/genes12050636 (PMC8146030; doi:10.3390/genes12050636)
Supplement: Supplementary file 1 [file genes-12-00636-s001.zip › reviewed_SupplementaryMaterials/File_S1.docx]

**First paleogenetic evidence from Middle Bronze Age human remains in Grotta della Monaca, Calabria (Southern Italy).**

**Francesco Fontani, Elisabetta Cilli, Fabiola Arena, Stefania Sarno, Alessandra Modi, Sara De Fanti, Adam Jon Andrews, Adriana Latorre, Paolo Abondio, Felice Larocca, Martina Lari, David Caramelli, Emanuela Gualdi-Russo and Donata Luiselli**

**File S1**

*Archaeological context and anthropological analyses*

Located 600m asl close to the Esaro River (Cosenza, north-western Calabria), Grotta della Monaca extends for 360 m in Triassic dolomitic limestone, developing into galleries, chambers, and narrow passages. It shows the presence of iron ore (limonite, lepidocrocite, goethite, hematite, yukonite) and copper ores (malachite, azurite, brochantite, libethenite, sampleite) [1–5]. The cave is conventionally divided into three main sectors: *Pregrotta* (the entrance gallery), *Sala dei Pipistrelli* (a very large hypogeum sector), *Cunicoli terminali* (two narrow tunnels in the innermost part of the cave) [6] (Figure 1).

The first survey of burial areas was conducted in 2000-2003 and allowed the discovery of twelve human skeletons. Out of these, only five have been diagnosed for sex due to the poor preservation conditions (4 males and 1 female) [7]. Further archaeological investigations, conducted until 2010, resulted in the finding of a mass grave with numerous human skeletal remains in a niche of the area of Cunicoli terminali (called *m5v*). The archaeological deposit was excavated using the “arbitrary level excavation method”, applied for mass grave burials in commingled contexts [8]. This archaeological sector was conventionally divided into three areas: *China* (the uppermost part of the deposit), *Sella* (an area of accumulation of commingled remains) and *Buca di L5* (the deepest part of the deposit). The remains were in a poor state of preservation due to several taphonomic factors. Among these factors, human frequentation has above all caused fragmentation of bones and their critical state of preservation [9]. In this area, the pottery assemblage is poor and commingled, but it was possible to recognize a typology of vessels used for funerary rituals, even if not directly attributable to the burials [10].

## *Osteological study*

The osteological remains buried in *m5v* of Grotta della Monaca were examined by traditional anthropological methodologies at the Laboratory of Archaeo-Anthropology and Forensic Anthropology of the Ferrara University (Italy). As suggested by protocols employed to reconstruct skeletons in collective and multiple burials [11–14], bones were grouped by typology (humerus, femur, tibia, etc.) and by side (right/left). The MNI (Minimum Number of Individuals) was assessed based on the anatomical features, considering bone recurrence, age-at-death, sex [15,16]. Skeletons were assembled by the articulation of bones, sex and age, specific features (diseases, bone robustness, muscle insertions, etc.). In compliance with the estimated age, we used the following categories [15] implemented with additional infant categories in accordance with Krenzer [17]: foetus (before birth), infant 1 (0 to 6 years old) (I1), infant 2 (7 to 12 years old) (I2), juvenile (12 to 20 years old) (J), young adult (20 to 35 years old) (YA), middle adult (35 to 50 years old) (MA), old adult (>50 years old) (OA. Age at death in subadults was estimated by dental eruption [18], epiphyseal fusion and dimensions of long bones [18–20], stage of bone ossification [21–24]. Cranial sutures [25,26], changes in the morphology of pubic symphyseal surface [27,28] and auricular surface [26,29], tooth wear [29,30] were used for the age-at-death assessment in adults. In adults, sex diagnosis was performed by the skull and pelvis morphology [15,25,31,32]. Whenever possible, the osteometric characters of the skull [33,34], mandible [35], scapula [36], humerus [24,31,37], radius [38], ulna [39], femur [37], tibia [31] and talus [40] have also been used for sex diagnosis. Although the method is considered less precise, sex was also assigned in subadults according to morphology of the mandible and ilium [41].

We examined macroscopically each specimen from a paleopathological point of view, detecting any osteological changes, and non-metric features. In particular, the presence of cribra cranii [42–44], cribra orbitalia (according to Knipp method reported by Brothwell, [30]), humeral and femoral cribra [45–47] have been investigated. Concerning the analysis of teeth, dental calculus was examined according to the scale of Brothwell [30] and successive modifications [48]. The degree of occlusal wear was scored according to Smith [49]. We recorded hypoplastic defects according to the standard published by Fédération Dentaire Internationale (FDI 1982). We also paid particular attention to the investigation of inherited features possibly present on teeth and skeletal remains.

The mass grave contained a minimum number of 24 individuals: 4 males, 6 females, and 14 undetermined. Regarding age at death, 75% of individuals were subadults (I1:7; I2:8; J:3; YA:4; OA:1; unknown:1).  Table S1 shows a summary of the main results concerning the osteological analysis of seven individuals (N. 5, 6, 7, 11, 12, 14, 24) involved in the molecular study. Porotic lesions were found to be the most common pathological condition: cribra were present in all these individuals although with different intensity and location of the affected areas.

The analysis of inherited skeletal traits led to the identification of some interesting markers among the human remains from the area *m5v*. In particular, three adult individuals show the congenital axis dysmorphism of the odontoid process – ossification is generally within five years. The attribution of these vertebrae to specific individuals was impossible as these vertebrae belonged to the same archaeological level (at the base of the Sella). From the comparison of the vertebrae with other specimens of known age at death, it resulted that they related to three young individuals with the first degree of expression of the pathology: the odontoid process is partially ossified, thus resulting in bifid dens (according to Aufderheide and Rodriguez-Martin[50]).

Carabelli’s tubercle, an additional dental cusp, was found on the first maxillary molar of individual 14. It is a well-formed cusp, corresponding to the 4th degree of expression following the classification of Borgognini Tarli and Pacciani [12]. We had seen several cases of this congenital trait on first molars in this necropolis, even if not associable to any of the other six individuals involved in the genetic analysis.

Human remains from Grotta della Monaca, as well as those of six other southern Italian prehistoric sites from Neolithic to Bronze Age, have also been recently analyzed through stable isotope analysis of bone collagen to reconstruct the diet of these agricultural-pastoral communities [51]. Inherited traits found in individuals from *m5v* have allowed us to advance the hypothesis of multiple family burials.  Other Italian Bronze Age sites corroborate this pattern. Specifically, multiple burial graves in the cave with related individuals were found at Grotta Manaccora in Apulia and at Grotta di Re Tiberio in Emilia-Romagna. In the latter, the remains of young women and newborn infants (0-3 months), probably interrelated, were found [52–57]. Moreover, several Middle Bronze Age burials -not in cave- involving family groups have been found at Toppo Daguzzo, Murgia Timone and Lavello in Basilicata, Ipogeo dei Bronzi (Madonna di Loreto, Trinitapoli) and Ipogeo degli Avori (Terra di Corte, San Ferdinando), Dolmen (Bisceglie), Santa Sabina (Carovigno) in Apulia [56,58].

Isotopic analysis of bone collagen [51] suggested that these communities had a varied diet consisting of terrestrial resources with a prevalent intake of vegetal proteins (cereals and legumes) and a modest intake of animal proteins for all the periods examined. During the Bronze Age, subsistence strategies were mainly based on cattle breeding rather than hunting. Some cereals (such as millet), already cultivated in north-central Italy, had not yet reached the south of the peninsula. As a result, a protein consumption of C_4_ plants-related was demonstrated for central northern Italy and C_3_ plants-related for southern Italy.

**References**

1. Dimuccio, L.A.; Garavelli, A.; Pinto, D.; Vurro, F.; Larocca, F. Le risorse minerarie. In *La miniera pre-protostoria di Grotta della Monaca (Sant’Agata di Esaro - Cosenza)*; Centro Regionale di Speleologia “Enzo dei Medici,” 2005; pp. 37–41.

2. Garavelli, A.; Pinto, D.; Vurro, F.; Mellini, M.; Viti, C.; Balic-Zunic, T.; Ventura, G.D. Yukonite from the Grotta Della Monaca Cave, Sant’Agata Di Esato, Italy: Characterization and Comparison with Cotype Material from the Daulton Mine, Yukon, Canada. *The Canadian Mineralogist* **2009**, *47*, 39–51, doi:10.3749/canmin.47.1.39.

3. Levato, C.; Larocca, F. The Prehistoric Iron Mine of Grotta Della Monaca (Calabria, Italy). *Anthropologica et Praehistorica* **2015**, *126*, 25–37.

4. Dimuccio, L.A.; Rodrigues, N.; Larocca, F.; Pratas, J.; Amado, A.M.; de Carvalho, L.A.E.B. Geochemical and Mineralogical Fingerprints to Distinguish the Exploited Ferruginous Mineralisations of Grotta Della Monaca (Calabria, Italy). *Spectrochimica Acta Part A: Molecular and Biomolecular Spectroscopy* **2017**, *173*, 704–720, doi:10.1016/j.saa.2016.10.021.

5. Caricola, I.; Breglia, F.; Larocca, F.; Hamon, C.; Lemorini, C.; Giligny, F. Prehistoric Exploitation of Minerals Resources. Experimentation and Use-Wear Analysis of Grooved Stone Tools from Grotta Della Monaca (Calabria, Italy). *Archaeol Anthropol Sci* **2020**, *12*, 259, doi:10.1007/s12520-020-01219-7.

6. Larocca, F. *La Miniera Pre-Protostorica Di Grotta Della Monaca (Sant’Agata Di Esaro - Cosenza)*; Centro Regionale di Speleologia “Enzo dei Medici,” 2005;

7. Scattarella, V.; Sublimi Saponetti, S.; Emanuel, P. Il sepolcreto ipogeo: aspetti antropologici. In *La miniera pre-protostorica di Grotta della Monaca (Sant’Agata di Esaro - Cosenza)*; Centro Regionale di Speleologia “Enzo dei Medici,” 2005; pp. 66–71.

8. Evis, L.H.; Hanson, I.; Cheetham, P.N. An Experimental Study of Two Grave Excavation Methods: Arbitrary Level Excavation and Stratigraphic Excavation. *STAR: Science & Technology of Archaeological Research* **2016**, *2*, 177–191, doi:10.1080/20548923.2016.1229916.

9. Arena, F.; Gualdi-Russo, E. Taphonomy and Post-Depositional Movements of a Bronze Age Mass Grave in the Archaeological Site of Grotta Della Monaca.; Annali dell’Università di Ferrara: Ferrara, September 10 2014.

10. Arena, F.; Larocca, F.; Onisto, N.; Gualdi-Russo, E. Il Sepolcreto Protostorico Di Grotta Della Monaca in Calabria. Aspetti Antropologici.; Annali dell’Università di Ferrara: Ferrara, November 11 2013.

11. Duday, H.; Courtaud, P.; Crubezy, E.; Sellier, P.; Tillier, A. L’Anthropologie « de Terrain » : Reconnaissance et Interprétation Des Gestes Funéraires. *Bulletins et Mémoires de la Société d’Anthropologie de Paris.* **1990**, 29–49, doi:10.3406/bmsap.1990.1740.

12. Pacciani, E. I procedimenti di restauro in laboratorio. In *I resti umani nello scavo archeologico. Metodiche di recupero e studio.*; Bulzoni, 1993.

13. Mallegni, F.; Paglialunga, L.; Ronco, D.; Vitello, A. Su Una Sepoltura Collettiva Di Bambini Di Epoca Tardo Medievale Rinvenuta Durante Lo Scavo Archeologico Di Piazza Dante a Pisa. *Rivista di Antropologia* **1994**, *72*, 119–134.

14. Cox, M.; Flavel, A.; Hanson, I.; Laver, J.; Wessling, R. *The Scientific Investigation of Mass Graves. Toward Protocols and Standard Operating Procedures*; Cambridge University Press: New York, 2008; ISBN 978-0-521-86587-6.

15. Buikstra, J.E.; Ubelaker, D.H. *Standards for Data Collection from Human Skeletal Remains. Proceedings of a Seminar at the Field Museum of Natural History*; Arkansas Archaeological Survey Research Series; Arkansas Archaeological Survey.; Fayetteville, Arkansas, 1994;

16. Lambacher, N.; Gerdau-Radonic, K.; Bonthorne, E.; Valle de Tarazaga Montero, F.J. Evaluating Three Methods to Estimate the Number of Individuals from a Commingled Context. *Journal of Archaeological Science: Reports* **2016**, *10*, 674–683, doi:10.1016/j.jasrep.2016.07.008.

17. Krenzer, U. *Compendio de métodos antropológico-forenses para la reconstrucción del perfil osteo-biológico.*; CAFCA, Centro de Analisis Forenses y Ciencias Aplicadas: Guatemala, 2006;

18. Ubelaker, D.H. *Human Skeletal Remains: Excavation, Analysis, Interpretation.*; Taraxacum: Washington DC, 1988;

19. Stloukal, M.; Hanakova, H. Die Lange Der Langsknochen Altslawischer Bevolkerungen Unter Besonderer Berucksichtigung von Wachstumfrager. *Homo* **1978**, *29*, 53–69.

20. Scheuer, L.; Black, S. *Developmental Juvenile Osteology*; Academic Press: New York, 2000;

21. Flecker, H. Time of Appearance and Fusion of Ossification Centers as Observed by Roentgenographic Methods. *AJR* **1942**, *47*, 97–159.

22. *The Malformed Fetus and Stillbirth: A Diagnostic Approach*; Winter, R.M., Knowles, S.A.S., Bieber, F.R., Baraitser, M., Eds.; Reprint.; John Wiley & Sons: Chichester, 1988; ISBN 978-0-471-90946-0.

23. Isçan, M.Y.; Kennedy, K.A.R. *Reconstructio of Life from the Skeleton.*; Alan R. Liss: New York, 1989;

24. France, D.L.; Horn, A.D. *Lab Manual and Workbook for Physical Anthropology*; West Publishing Company, 1992; ISBN 978-0-314-93445-1.

25. Acsádi, G.; Nemeskéri, J. *History of Human Life Span and Mortality.*; Akadémiai Kiadó: Budapest, 1970;

26. Meindl, R.S.; Lovejoy, C.O. Ectocranial Suture Closure: A Revised Method for the Determination of Skeletal Age at Death Based on the Lateral-Anterior Sutures. *Am J Phys Anthropol* **1985**, *68*, 57–66, doi:10.1002/ajpa.1330680106.

27. Todd, T.W. Age Changes in the Pubic Bone. III: The Pubis of the White Female. IV: The Pubis of the Female White-Negro Hybrid. *American Journal of Physical Anthropology* **1921**, *4*, 1–70, doi:https://doi.org/10.1002/ajpa.1330040102.

28. Brooks, S.; Suchey, J.M. Skeletal Age Determination Based on the Os Pubis: A Comparison of the Acsádi-Nemeskéri and Suchey-Brooks Methods. *Hum. Evol.* **1990**, *5*, 227–238, doi:10.1007/BF02437238.

29. Lovejoy, C.O.; Meindl, R.S.; Pryzbeck, T.R.; Mensforth, R.P. Chronological Metamorphosis of the Auricular Surface of the Ilium: A New Method for the Determination of Adult Skeletal Age at Death. *Am J Phys Anthropol* **1985**, *68*, 15–28, doi:10.1002/ajpa.1330680103.

30. Brothwell, D.R. *Digging Up Bones*; Oxford University Press, 1981;

31. Ferembach, D.; Schwindezky, I.; Stoukal, M. Recommendation for Age and Sex Diagnoses of Skeletons. *Journal of Human Evolution* **1980**, *9*, 517–549.

32. Krogman, W.M.; Isçan, M.Y. *The Human Skeleton in Forensic Medicine.*; Charles C. Thomas: Springfield, Illinois, 1986;

33. Giles, E. Sex Determination by Discriminant Function Analysis of the Mandible. *Am J Phys Anthropol* **1964**, *22*, 129–135, doi:10.1002/ajpa.1330220212.

34. Demoulin, F. Importance de Certaines Mesures Crâniennes (En Particulier de La Longueur Sagittale de La Mastoide) Dans La Determination Sexuelle Des Crânes. **1972**, *Bulletins et Mémoires de la Société d’Anthropologie de Paris.*, 259–264.

35. Piquet, M.-M. Étude sur la robustesse de la mandibule. *Bulletins et Mémoires de la Société d’Anthropologie de Paris* **1956**, *7*, 204–224, doi:10.3406/bmsap.1956.9725.

36. Olivier, G.; Pineau, H. Détermination de l’âge Du Foetus et de l’embryon. *Archives d’Anatomie* **1958**, *5*, 67–88.

37. Dittrick, J.; Suchey, J.M. Sex Determination of Prehistoric Central California Skeletal Remains Using Discriminant Analysis of the Femur and Humerus. *Am J Phys Anthropol* **1986**, *70*, 3–9, doi:10.1002/ajpa.1330700103.

38. Cattaneo, C.; Grandi, M.A. *Antropologia e Odontologia Forense: Guida Allo Studio Dei Resti Umani.*; Monduzzi: Bologna, 2004; ISBN 88-323-4104-2.

39. France, D.L. Observational and metric analysis of sex in the skeleton. In *Forensic Osteology: Advances in the Identification of Human Remains*; Charles C. Thomas: Springfield, Illinois, 1998; pp. 163–186.

40. Gualdi-Russo, E. Sex Determination from the Talus and Calcaneus Measurements. *Forensic Sci Int* **2007**, *171*, 151–156, doi:10.1016/j.forsciint.2006.10.014.

41. Schutkowski, H. Sex Determination of Infant and Juvenile Skeletons: I. Morphognostic Features. *Am J Phys Anthropol* **1993**, *90*, 199–205, doi:10.1002/ajpa.1330900206.

42. Stuart‐Macadam, P. Porotic Hyperostosis: Representative of a Childhood Condition. *American Journal of Physical Anthropology* **1985**, *66*, 391–398, doi:https://doi.org/10.1002/ajpa.1330660407.

43. Schultz, M. Paleohistopathology of Bone: A New Approach to the Study of Ancient Diseases. *Am J Phys Anthropol* **2001**, *Suppl 33*, 106–147, doi:10.1002/ajpa.10024.abs.

44. Rinaldo, N.; Zedda, N.; Bramanti, B.; Rosa, I.; Gualdi-Russo, E. How Reliable Is the Assessment of Porotic Hyperostosis and Cribra Orbitalia in Skeletal Human Remains? A Methodological Approach for Quantitative Verification by Means of a New Evaluation Form. *Archaeol Anthropol Sci* **2019**, *11*, 3549–3559, doi:10.1007/s12520-019-00780-0.

45. Nathan, H.; Haas, N. “Cribra Orbitalia”. A Bone Condition of the Orbit of Unknown Nature. Anatomical Study with Etiological Considerations. *Isr J Med Sci* **1966**, *2*, 171–191.

46. Miquel-Feucht, M.J.; Polo-Cerdá, M.; Villalaín-Blanco, J.D. Cribra Orbitalia vs. Cribra Femora: New Contributions to the Cribose Syndrome. *Journal of Paleopathology* **1999a**, *11*, 84.

47. Miquel-Feucht, M.J.; Polo-Cerdá, M.; Villalaín-Blanco, J.D. Anthropological and Paleopathological Studies of a Mass Execution during the War of Independence in Valencia, Spain (1808-1812). *11*, 15–23.

48. Belcastro, M.G.; Mariotti, V.; Facchini, F.; Bonfiglioli, B. Proposal of a Data Collection Form to Record Dento-Alveolar Features--Application to Two Roman Skeletal Samples from Italy. *Coll Antropol* **2004**, *28*, 161–177.

49. Smith, B.H. Patterns of Molar Wear in Hunger-Gatherers and Agriculturalists. *Am J Phys Anthropol* **1984**, *63*, 39–56, doi:10.1002/ajpa.1330630107.

50. Aufderheide, A.C.; Rodriguez-Martin, C. *Human Paleopathology.*; Cambridge University Press, 1998; ISBN 052155203.

51. Arena, F.; Gualdi-Russo, E.; Olsen, J.; Philippsen, B.; Mannino, M.A. New Data on Agro-Pastoral Diets in Southern Italy from the Neolithic to the Bronze Age. *Archaeol Anthropol Sci* **2020**, *12*, 245, doi:10.1007/s12520-020-01209-9.

52. Cremonesi, G. Contributo Alla Conoscenza Della Preistoria Del Fucino: La Grotta Di Ortucchio e La Grotta La Punta. **1968**, *Rivista di scienze preistoriche*, 145–204.

53. Cremonesi, G. La Grotta dell’orso di Sarteano. I livelli dell’età dei metalli. *Origini. Preistoria e protostoria delle civiltà antiche.* **1968**, 247–331.

54. Pacciarelli, M. *Acque, Grotte e Dei. 3000 Anni Di Culti Preromani in Romagna, Marche e Abruzzo.*; Musei Civici di Imola, 1997;

55. Gullì, D. Primo Convegno Di Archeologia Di Sciacca. Incontro Di Studi Preistorici in Memoria Di Santo Tiné.; Sciacca, 2011.

56. Bietti Sestieri, A.M. *L’Italia nell’età del bronzo e del ferro. Dalle palafitte a Romolo (2200-700 a.C.)*; Carocci editore, 2010; ISBN 88-430-5207-1.

57. Gullì, D. L’occupazione delle grotte in età preistorica nel territorio agrigentino. In *Diffusione delle conoscenze: Atti del XXI Congresso Nazionale di Speleologia, Trieste, 2-5 giugno 2011.*; EUT Edizione Università di Trieste: Trieste, 2013; pp. 258–267.

58. Tunzi Sisto, A.M. *Ipogei Della Daunia. Preistoria Di Un Territorio*; Grenzi, 1999;
