# Supplementary figures and images for: First Bronze Age Human Mitogenomes from Calabria (Grotta Della Monaca, Southern Italy)
Source: Genes (Basel). 2021 Apr 25;12(5):636. doi: 10.3390/genes12050636 (PMC8146030; doi:10.3390/genes12050636)

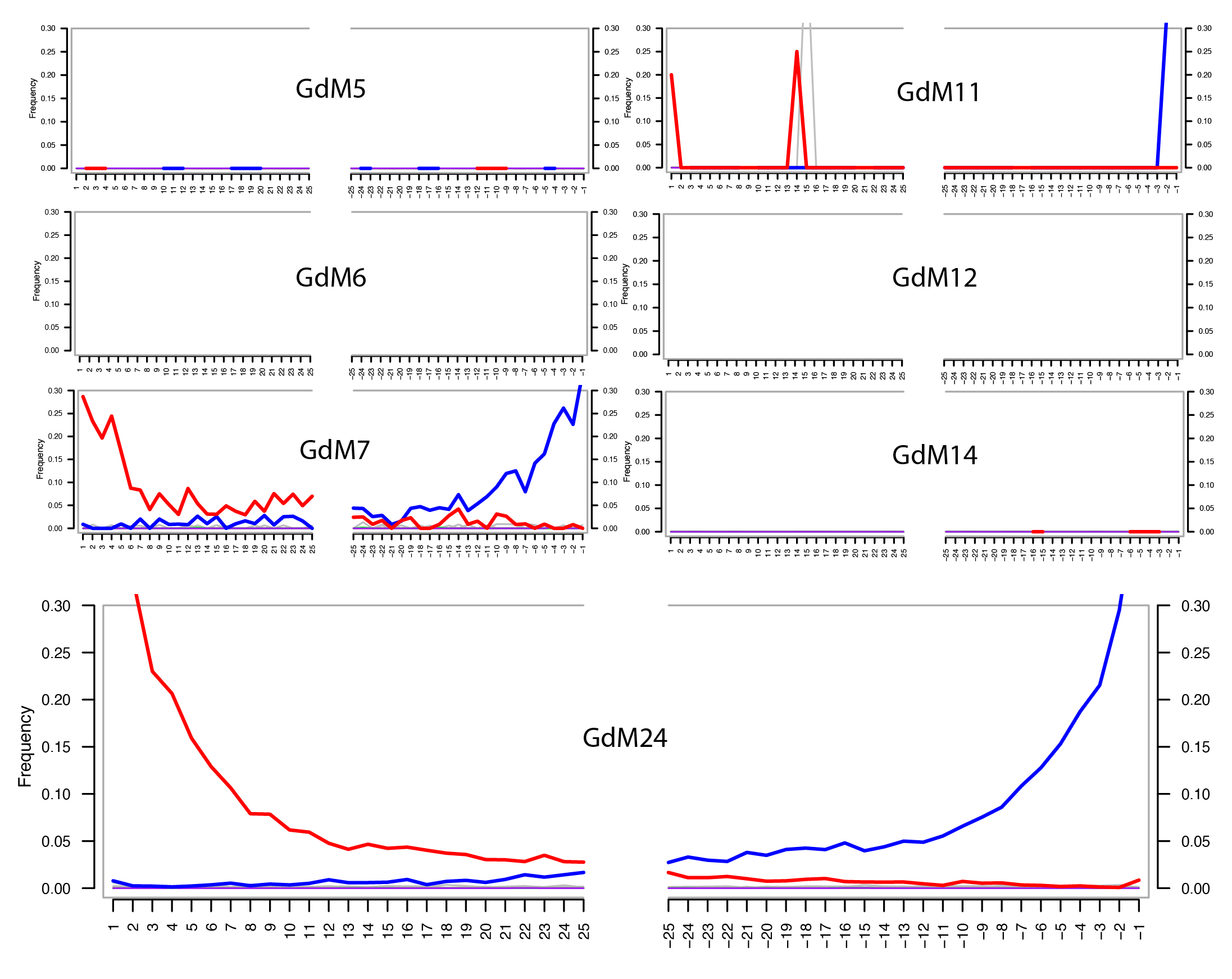

Supplement: Supplementary file 1 [file genes-12-00636-s001.zip › reviewed_SupplementaryMaterials/Fig_S2.png]

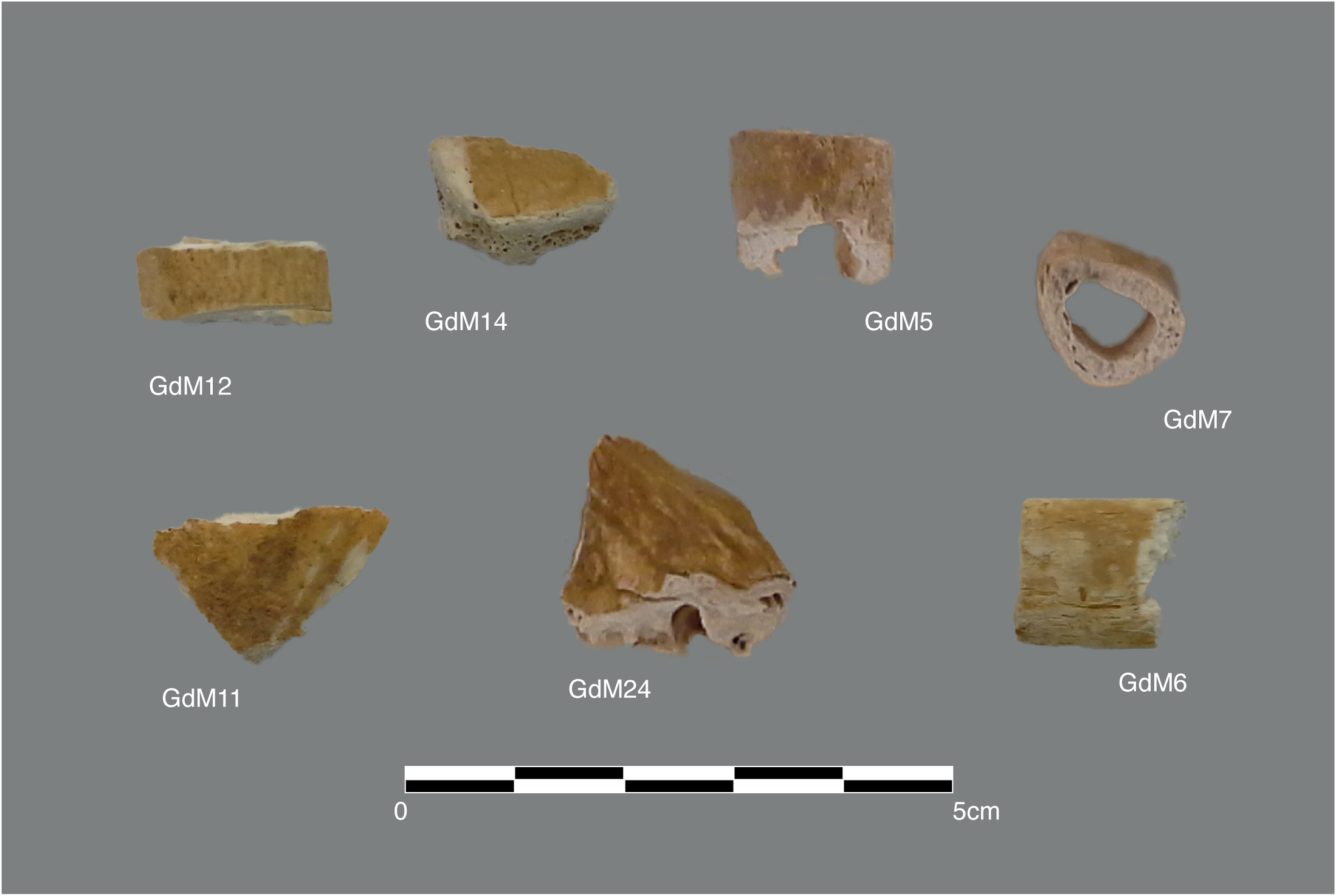

Supplement: Supplementary file 1 [file genes-12-00636-s001.zip › reviewed_SupplementaryMaterials/Fig_S1.tif]
